# Capsaicin and Piperine Can Overcome Multidrug Resistance in Cancer Cells to Doxorubicin

**DOI:** 10.3390/molecules23030557

**Published:** 2018-03-02

**Authors:** Hanmei Li, Sonja Krstin, Shihui Wang, Michael Wink

**Affiliations:** 1 Institute of Pharmacy and Molecular Biotechnology, Heidelberg University, Im Neuenheimer Feld 364, D-69120 Heidelberg, Germany; hanmei.li@outlook.com (H.L.); krstin@uni-heidelberg.de (S.K.); 2 Heidelberg University Biochemistry Centre (BZH), Heidelberg University, Im Neuenheimer Feld 328, 69120 Heidelberg, Germany; wangsh219@yahoo.com

**Keywords:** capsaicin, piperine, multidrug resistance, Caco-2, HCT 116, CEM/ADR 5000, CCRF-CEM

## Abstract

Background: Multidrug resistance (MDR) can develop in cancer cells after treatment with anticancer drugs, mainly due to the overexpression of the ATP-binding cassette (ABC) transporters. We analyzed the ability of two pungent-tasting alkaloids—capsaicin and piperine from *Capsicum frutescens* and *Piper nigrum*, respectively—to reverse multidrug resistance in the cancer cell lines Caco-2 and CEM/ADR 5000, which overexpress P-glycoprotein (P-gp) and other ABC transporters. Methods: The MTT assay was first used to determine the cytotoxicity of doxorubicin, the alkaloids, and digitonin alone, and then their combinations. Furthermore, rhodamine (Rho) 123 and calcein-AM were used to detect the effects of alkaloids on the activity of P-gp. Results: Capsaicin and piperine synergistically enhanced the cytotoxicity of doxorubicin in Caco-2 and CEM/ADR 5000 cells. Furthermore, capsaicin and piperine increased the intracellular accumulation of the fluorescent P-glycoprotein (P-gp) substrates rhodamine and calcein and inhibited their efflux from the MDR cell lines. Conclusion: Our study has demonstrated that capsaicin and piperine are P-gp substrates and have potential chemosensitizing activity, which might be interesting for the development of novel modulators of multidrug resistance.

## 1. Introduction

Cancer is a major cause of death worldwide. It is characterized by uncontrolled cell division, dedifferentiation of a normal cell, and metastatic growth. Researchers have concluded that tumorigenesis in humans is a multistep process with genetic alterations that drive the transformation of normal human cells into highly malignant derivatives [1]. Anticancer drugs, such as doxorubicin, vinblastine, vincristine, epirubicin, etoposide, and imatinib, are widely used in chemotherapy [2].

Despite many side effects, chemotherapeutics are still used for the effective treatment of metastatic tumors. However, the development of resistance to anticancer drugs is a common clinical issue in the treatment of cancer patients. The ability of cancer cells to become simultaneously resistant to different structurally unrelated drugs, a trait known as multidrug resistance, remains a major problem associated with the curative treatment of cancer patients [3].

Multidrug resistance is the phenomenon by which cancer cells, after being exposed to one anticancer drug, develop resistance to various other anticancer drugs that are structurally and functionally different from the initial drug. This resistance, intrinsic or acquired, can be attributed to drug efflux and drug metabolism [4]. Drug efflux depends on transporters that are located in the outer biomembrane of cancer cells. These transmembrane proteins belong to the class of ATP-binding cassette (ABC) transporters that eliminate xenobiotics from cells into bile, urine or feces by using the energy released from ATP hydrolysis. This leads to a reduction in the bioavailability of drugs [5]. Forty-nine *ABC* transporter genes have been identified in the human genome and are arranged into seven subfamilies designated from A to G [6]. The most typical efflux transmembrane protein is P-glycoprotein (P-gp) with a molecular weight of 170 kDa. 

Once multidrug resistance (MDR) develops, chemotherapy is no longer effective, even when high doses of drugs are used. This leads to toxic effects and stimulation of further resistance. These problems could be addressed by the use of other anticancer drugs, which bypass the resistance mechanism, or by co-administration of substances that inhibit ABC transporters. Such agents are known as chemosensitizers since they can reverse resistance to anticancer drugs and eventually re-sensitize the cancer cells to anticancer drugs [7]. Secondary metabolites, such as alkaloids, phenolics, and terpenoids, can function as substrates or inhibitors of P-gp and could be administered as chemosensitizers in combination with a cytotoxic agent, for example, doxorubicin [8].

Cellular biomembranes are selectively permeable to ions and organic molecules, controlling the movement of the substances in and out of cells. Digitonin, which has many applications in biochemistry, is a steroidal saponin [9] that interacts with biomembranes rich in cholesterol. Digitonin can permeabilize cell membranes [10,11] and is also a P-gp substrate. Our previous studies have shown that digitonin can synergistically enhance cytotoxicity in combination with other secondary metabolites and doxorubicin [12,13]. 

**Figure 1:**
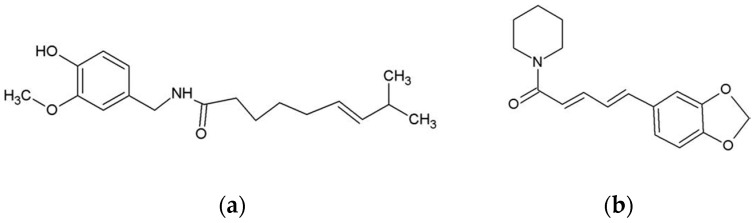
Chemical structures of (**a**) capsaicin and (**b**) piperine.

Capsaicin and piperine are pungent alkaloids of *Capsicum frutescens* and of *Piper nigrum*, respectively. They are widely used as spices in many cultures. Their chemical structures are presented in Figure 1. Capsaicin has been reported to exhibit chemopreventive effects, which suppress carcinogenesis of skin, lung, colon, tongue, and prostate [14], while piperine also has potential as a new anticancer drug [15].

In this study, we wanted to explore whether these two secondary metabolites have the ability to inhibit the activity of P-gp in the multidrug-resistant cancer cell lines Caco-2 and CEM/ADR 5000 cells when combined with doxorubicin. A two-drug combination of the alkaloids with doxorubicin and a three-drug combination of the alkaloids plus digitonin plus doxorubicin were used to evaluate the presence of synergy. Furthermore, a P-gp transporter activity assay was used to detect whether capsaicin and piperine inhibit the activity of P-gp. Two other cancer cell lines, HCT 116 and CCRF-CEM, which do not overexpress P-gp, were used as controls.

## 2. Results

### 2.1. Cytotoxicity of Capsaicin and Piperine in Different Cancer Cell Lines

The cytotoxicity of capsaicin and piperine was investigated using four different cell lines (MDR: Caco-2, CEM/ADR 5000; and non-MDR: HCT 116, CCRF-CEM). Caco-2 and HCT 116 are heterogeneous human epithelial colorectal adenocarcinoma cells and Caco-2 can highly express P-gp on its apical surface [16]. CCRF-CEM is an acute lymphoblastic leukemia cell line. CEM/ADR 5000 is derived from CCRF-CEM and overexpresses P-gp [17]. The two compounds showed cytotoxic effects against human colon and leukemia cell lines; however, their cytotoxicity was much lower than that of doxorubicin (Table 1). HCT 116 and CCRF-CEM cells were more sensitive to capsaicin and piperine than the P-gp overexpressing Caco-2 and CEM/ADR 5000 cell lines, implying that P-gp may be involved in the resistance mechanism.

### 2.2. Capsaicin and Piperine Increased the Sensitivity of MDR Cancer Cells to Doxorubicin

**Figure 2:**
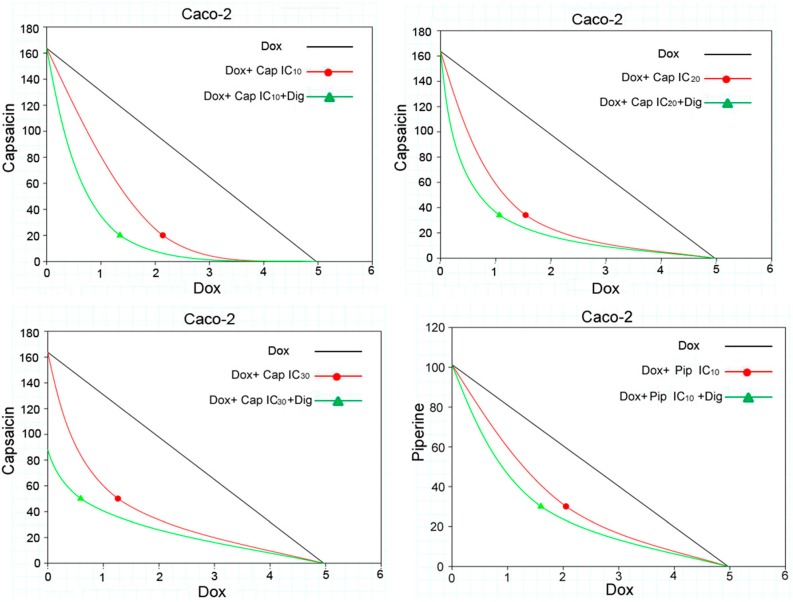

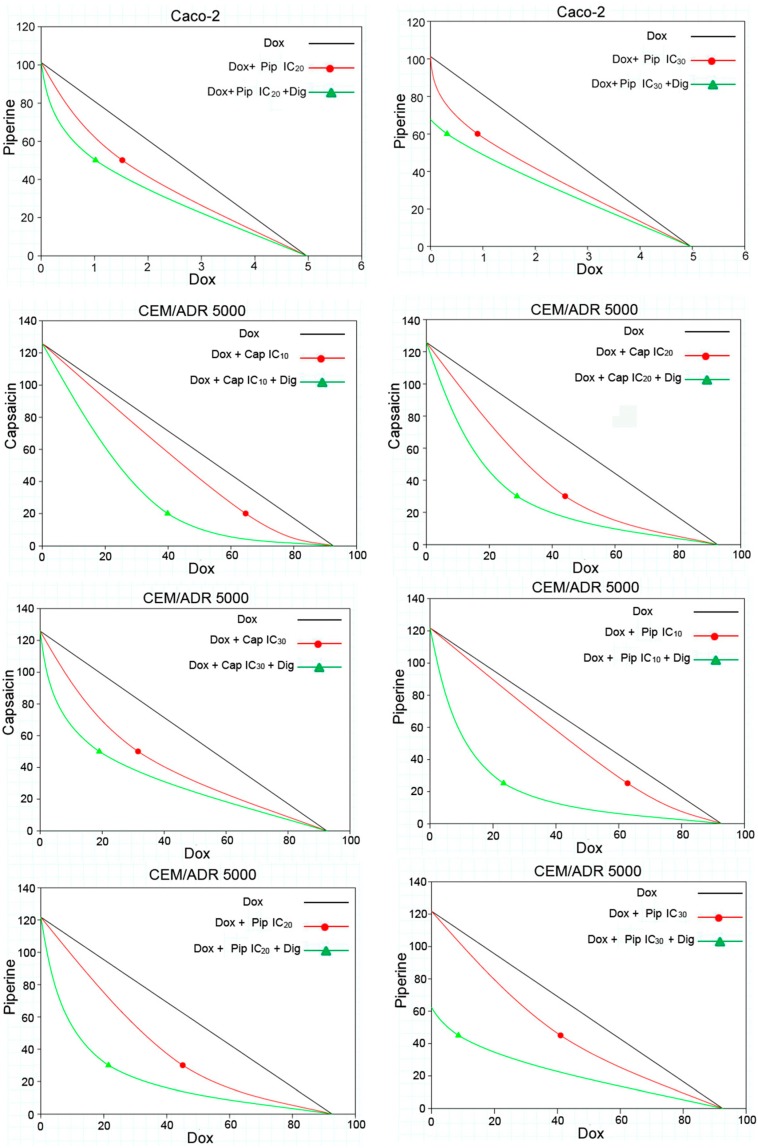
Isobologram analysis of the drugs’ interactions. The IC_50_ concentration of doxorubicin is set on the *x*-axis and the IC_50_ of the secondary metabolitesn the *y*-axis. The line connecting these two points means additivity. Points below the line indicate synergy. Dox—doxorubicin; Cap—capsaicin; Pip—piperine; Dig—digitonine.

Based on the IC_50_ values determined above, three non-toxic concentrations (IC_10_, IC_20_, and IC_30_) of capsaicin and piperine were combined with doxorubicin. In addition, a three-drug combination was designed in which the saponin digitonin was added as a third partner. The IC_50_ values of doxorubicin alone, in two-drug combinations, or in three-drug combinations in Caco-2 and CEM/ADR 5000 cell lines are recorded in Table 2 and Table 3, respectively. Furthermore, the combination indexes (CI) and the dose reduction indexes (DRI) were analyzed for two- and three-drug combinations. An isobologram analysis was also employed to analyze the nature of the interaction (Figure 2).

As shown in Table 2 and Table 3, capsaicin and piperine can significantly decrease the IC_50_ value of doxorubicin, thus showing chemosensitizing activity. The nature of the combinations was evaluated by CI analysis, as described before [18]. In general, CI values <1 signify synergism; =1, additive effects; and >1, antagonism. All the CI values calculated are below 1, meaning that the interactions are synergistic. Digitonin can enhance this synergism even further. For example, in Caco-2, the combination of piperine (IC_30_) and doxorubicin resulted in moderate synergism (+ +). When digitonin was added (0.5 μM for Caco-2 and CEM/ADR 5000), the synergism was enhanced (+ + +). Moreover, capsaicin and piperine had the same effects in CEM/ADR 5000. Compared to two-drug combinations, digitonin remarkably increased the synergism in both MDR cell lines.

The dose reduction indexes (DRI) were calculated as described by Krstin et al. (2015) [19]. Preferable DRI values would be >1, indicating a reduced dose to maintain or increase the therapeutic efficacy. As shown in Table 2 and Table 3, all the DRI values are >1. However, values above 1 do not necessarily indicate synergism because additive effects, or even slight antagonism, can also lead to values >1 [19]. DRI alone is not adequate to determine whether an interaction is synergistic or not. Combination indexes (CI) and isobologram analysis are also needed to achieve an accurate conclusion.

The results of the isobologram analysis are shown in Table 2 and Table 3 and Figure 2. The IC_50_ concentrations of doxorubicin and capsaicin or piperine are plotted on the *x-* or *y-*axis corresponding to (C_Dox_, 0) and (0, C_SM_), respectively. The line connecting these two points means additivity. The concentrations of doxorubicin and secondary metabolites used in combination to reach the same effect (IC_50_) denoted as (C_Dox_, C_SM_) are placed in the same plot. As shown in Figure 2, all the (C_Dox_, C_SM_) are below the additivity line indicating that the two-drug combinations and three-drug combinations resulted in synergism.

### 2.3. Capsaicin and Piperine Inhibited the Activity of P-gp

**Figure 3:**
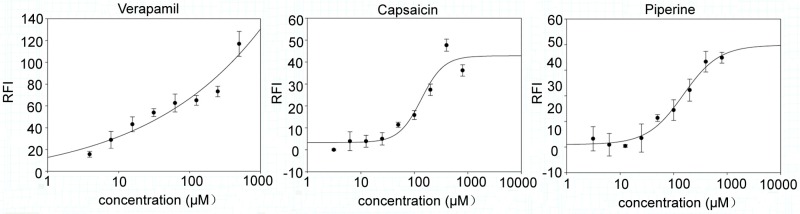
Effects of alkaloids and the positive control with verapamil on rhodamine (Rho) 123 retention in Caco-2 cells. Cells treated with DMSO were used as a solvent control. Data are presented as the mean ± SD.

In Caco-2 cells, the effect of capsaicin and piperine on P-gp activity was assessed by measuring the intracellular accumulation of rhodamine (Rho) 123. As shown in Figure 3, Caco-2 cells exhibited a notable increase in Rho 123 fluorescence in a dose-dependent manner when treated with capsaicin and piperine, implying that the activity of P-gp is affected by these two drugs.

**Figure 4:**
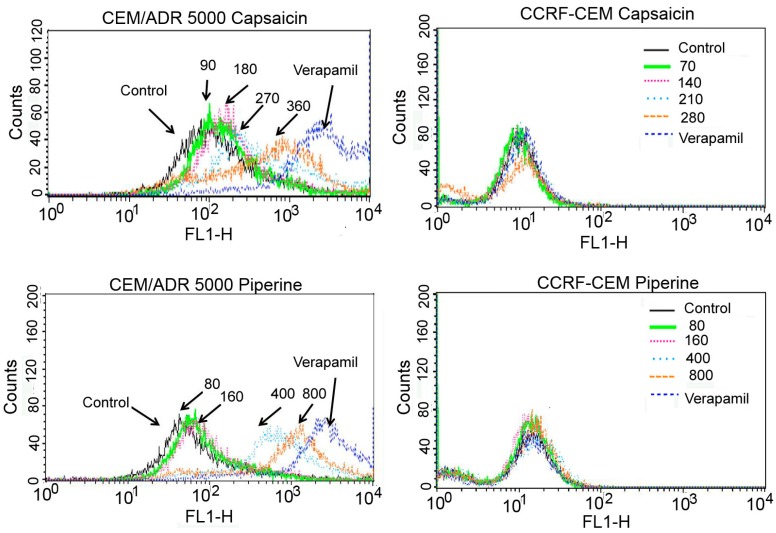
Histograms of flow cytometry of calcein accumulation in CEM/ADR 5000 and CCRF-CEM cells. Cells treated with DMSO were used as the negative control. The numbers drawn in this graph mean concentrations (μM). Cells treated with 20 μM verapamil were used as the positive control.

Flow cytometry was used to detect fluorescence of calcein (as explained in the methods section). The concentrations used here were based on the IC_50_ of the drug in the respective cell line. The concentrations used were described in Figure 4. Verapamil (20 μM) was used as a positive control. As shown in Figure 4, compared to the negative control (treated with DMSO, below 0.1%), capsaicin and piperine shifted the fluorescence intensity of calcein rightwards in a concentration-dependent manner, indicating that capsaicin and piperine can inhibit the activity of P-gp in resistant leukemia cells. In CCRF-CEM cells, the fluorescence intensity showed no variation compared to the negative control. The results demonstrate that capsaicin and piperine increased the retention of calcein in the CEM/ADR 5000 cells by affecting the activity of P-gp.

## 3. Discussion

MDR in cancer cells is mostly due to the overexpression of ABC transporters in the cell membrane, which effluxes chemotherapeutical drugs out of cells [7]. To address MDR, many modulators of ABC transporters have been investigated, especially secondary metabolites from plants [8]. Furthermore, several natural compounds that affect ABC transporters have been investigated and summarized [14]. Both capsaicin and piperine are dietary natural products. Capsaicin from chili not only exhibits cancer preventive properties by inhibiting the activity of NF-κB [20] but also plays a beneficial role in overcoming obesity and cardiovascular and gastrointestinal conditions [21]. Piperine from black pepper is widely used as a spice but possesses a variety of medicinal properties [22,23,24]. Our study demonstrated that capsaicin and piperine had a notable reversal effect in P-gp overexpressing cell lines, Caco-2 and CEM/ADR 5000. They significantly and synergistically increased the cytotoxicity of doxorubicin.

Cell membranes are impermeable to polar or charged molecules. Digitonin, which can disturb the membrane stability and enhance its permeability, was used in our study to increase the cytotoxicity of doxorubicin. In the three-drug combination, the synergistic effect was enhanced after adding digitonin. This method was used in our lab before; the assumption is that digitonin can increase the uptake of polar cytotoxic secondary metabolites, which are unable to enter cells by free diffusion [13].

The current study investigated the combination of two secondary metabolites (capsaicin and piperine) and the intercalating agent doxorubicin in drug-resistant colon and leukemia cells. We included two cell lines without P-gp overexpression (i.e., HCT 116 and CCRF-CEM) that are more sensitive to piperine, capsaicin, and digitonin than Caco-2 and CEM/ADR 5000 cells. In addition, with the rhodamine and calcein assay, we showed that piperine and capsaicin competed with the fluorescent substrates of P-gp. Thus, we consider that both alkaloids are potential substrates of P-gp, which would explain how they can sensitize these cancer cells to doxorubicin. The experiments with digitonin were added to test whether the synergism could be enhanced by adding a second P-gp substrate, as done in former papers [13].

Three methods were used to evaluate the drug interactions. Combination indexes (CI), isobolograms, and dose reduction indexes (DRI) were employed to detect whether interactions were synergistic or additive. Synergism was observed in two-combination and three-combination treatments in the drug-resistant cells (i.e., Caco-2 and CEM/ADR 5000 cells). To understand the mechanism of the reversal activity, the inhibition effect of capsaicin and piperine on Caco-2 and CEM/ADR 5000 cells was assessed. Consistent with the results of Li et al. and Nebekura et al. where capsaicin and piperine had inhibitory effects on human P-gp [25,26], our results clearly show that capsaicin and piperine can strongly inhibit the intracellular efflux of Rho 123 from Caco-2 cells and of calcein from CEM/ADR 5000 cells. However, no effect was observed (as expected) in the sensitive CCRF-CEM cell line.

There are two suggested mechanisms for the inhibition of ABC transporter activity by secondary metabolites. (I) The secondary metabolites are the substrates of the ABC transporters such as Rho 123 and thus competitive inhibitors. As a consequence, the ABC transporters pump out the secondary metabolites instead of the anticancer drugs, for example, doxorubicin, resulting in a higher concentration of anticancer drugs inside the cells and therefore improving the efficacy of the anticancer drugs. Many alkaloids inhibit the activity of ABC transporters by using this mechanism. (II) The secondary metabolites are inhibitors of ABC transporter proteins. They bind to the ABC transporters reversibly or irreversibly, thus reducing the activity of ABC transporters. Polyphenols, which affect most proteins, use this mechanism to inhibit the activity of the ABC transporters [27]. Since capsaicin and piperine are alkaloids, they apparently inhibit the activity of P-gp by using the first mechanism.

As dietary phytochemicals, capsaicin and piperine have no or very few toxic side effects. Some researchers have reported that capsaicin has the ability to alleviate osteoarthritis pain and reduce cough [28,29]. Administration of capsaicin may reduce the metastatic burden in transgenic mice with adenocarcinoma of the prostate [30]. Piperine exhibits many functions, for example, antidepressant, hepatoprotective, anti-metastatic, anti-thyroid, antitumor, and anti-inflammatory activities [31,32]. In addition to the listed beneficial abilities, our results also demonstrate the multidrug resistance reversal activity of capsaicin and piperine, which is mediated by their modulation of P-gp. This implies that they could make excellent candidates as reversal modulators. However, such an application needs more evidence from in vivo studies.

## 4. Materials and Methods

### 4.1. Chemicals

Capsaicin, piperine, calcein-AM (CAM), rhodamine 123 (Rho 123), verapamil hydrochloride, digitonin, fetal bovine serum (FBS), dimethyl sulfoxide (DMSO), and 3-(4,5-dimethylthiazol-2-yl)-2,5-diphenyl-tetrazolium bromide (MTT) were purchased from Sigma-Aldrich GmbH, Steinheim, Germany. RPMI-1640, DMEM, non-essential amino acids (NEAA), penicillin–streptomycin, sodium pyruvate, trypsin–EDTA, and l-glutamine came from Gibco, Karlsruhe, Germany.

### 4.2. Cell Culture

Human T-cell lymphoma CCRF-CEM cells and the derived doxorubicin-resistant subline CEM/ADR 5000, which overexpress P-gp, were cultured in RPMI 1640 medium supplemented with 10% (*v*/*v*) FBS, 2 mM l-glutamine, and 100 U/mL penicillin, and 100 μg/mL streptomycin. The adherent human colon cancer cell line HCT 116 and Caco-2 were maintained in DMEM supplemented with 10% (*v*/*v*) FBS, 2 mM l-glutamine, 100 U/mL penicillin, and 100 μg/mL streptomycin. In addition, 1 mM sodium pyruvate and 1% (*v*/*v*) NEAA were added to Caco-2 cell medium. HCT 116 and Caco-2 cells were detached from the culture vessel by adding trypsin–EDTA for 5 min. Cells were cultivated at 37 °C, 5% CO_2_, and 95% humidity. All experiments were performed with cells in the logarithmic growth phase.

### 4.3. Cytotoxicity Assays

Cell viability was determined using the conventional MTT assay [33]. Briefly, 5 × 10^3^ Caco-2 or HCT 116 cells/well were seeded in 96-well plates and incubated overnight. Different concentrations of capsaicin and piperine were then added and incubated for 48 h. Afterwards, MTT (0.5 mg/mL) was pipetted into each well and incubated for 2–4 h at 37 °C. The produced formazan crystals were dissolved in 100 μL DMSO and the optical density was measured at 570 nm using a Tecan microplate reader (Crailsheim, Germany). For CCRF-CEM and CEM/ADR 5000 cell lines, 2 × 10^4^ cells/well were treated with drugs for 48 h. Each compound was analyzed independently at least three times. IC_50_ is the drug concentration that kills 50% of the cells. IC_50_ values were determined by SigmaPlot 11.0 software (Systat Software Inc., San Jose, CA, USA).

### 4.4. Drug Combination Assays

For the two-drug combinations, three non-toxic concentrations (IC_10_, IC_20_, and IC_30_) of capsaicin and piperine were used in combination with doxorubicin (Sigma-Aldrich GmbH, Steinheim, Germany) to measure whether these alkaloids could increase the sensitivity of MDR cancer cells toward doxorubicin. Briefly, Caco-2 and CEM/ADR 5000 cells were seeded into 96-well plates and incubated with serial dilutions of doxorubicin combined with a fixed non-toxic concentration of alkaloid as mentioned above. An MTT assay was carried out as described above to detect the cytotoxicity of doxorubicin alone and in combination with the alkaloids against Caco-2 and CEM/ADR 5000 cells.

For the three-drug combinations, doxorubicin was combined with an alkaloid of three different concentrations (IC_10_, IC_20_, IC_30_) plus a fixed non-toxic concentration of digitonin (0.5 μM). Briefly, Caco-2 and CEM/ADR 5000 cells were seeded into 96-well plates and incubated with serial dilutions of doxorubicin and fixed concentrations of alkaloids plus digitonin. The cytotoxicity was determined using the MTT assay as described above.

### 4.5. Analysis of Combination Effects

In order to analyze whether the combinations resulted in synergism, additivity, or antagonism, the combination indexes (CI) method, isobolograms, and dose reduction indexes (DRI) were employed [34]. The procedure has already been described in Eid et al. (2012) [12].

### 4.6. Activity of ABC Transporters

The effects of capsaicin and piperine on MDR activity in Caco-2 cells were determined by incubating the cells with a fluorescent P-gp substrate Rho 123, which is readily effluxed. Briefly, Caco-2 cells were seeded in a 96-well transparent plate and incubated at 37 °C until a confluent monolayer was formed. Usually, this took 4–6 days. Then, the cells were treated with various concentrations of capsaicin and piperine for 4 h at 37 °C. Rho 123 (final concentration 10 μM) was added and the cells were incubated for a further 90 min. After that, the cells were washed twice with 1 × PBS, and then the wells were filled with washing buffer (0.1% Triton X-100 in 1 × PBS) and kept on ice in the dark before measurement. Cells treated with DMSO (below 0.1%) were used as a negative control. A Tecan reader (Crailsheim, Germany) was used to measure the fluorescence intensity of Rho 123 at excitation/emission wavelengths of 485/535 nm. The relative fluorescence intensity (RFI) of treated cells was calculated as follows:

(1) $$\mathrm{RFI}=\frac{{\mathrm{RFI}}_{\mathrm{SM}}-{\mathrm{RFI}}_{\mathrm{control}}}{{\mathrm{RFI}}_{\mathrm{control}}}\times 100\%$$

For CEM/ADR 5000 and CCRF-CEM suspension cells, calcein-AM (CAM) was used. CAM is converted by esterases into calcein, resulting in fluorescence after complexing with calcium ions. Briefly, 1 × 10^4^ cells/mL were treated with different concentrations of the alkaloids. After incubation for 90 min at 37 °C, CAM (150 nM for CEM/ADR 5000, 75 nM for CCRF-CEM) was added and incubated for a further 90 min. The cells were collected and washed twice with 1 × PBS. Fluorescence was measured by a FACS^®^Calibur instrument (Becton Dickinson, Franklin Lakes, NJ, USA). At least 10,000 cells per sample were counted and acquired through the FL1 channel. BD Cell Quest pro software (Becton Dickinson, Franklin Lakes, NJ, USA) was used for data analysis. Verapamil, a known P-gp inhibitor, was used as a positive control. 

### 4.7. Statistical Analysis

All experiments were carried out at least three times. The IC_10_, IC_20_ and IC_30_ values of each compound were determined using the online software Calculate ECanything from EC50-GraphPad (Graphpad Software, San Diego, CA, USA). Data analysis was performed with GraphPad Prism 6.0 (Graphpad Software, San Diego, CA, USA). Data are presented as the mean ± SD. Isobologram analysis and the IC_50_ were calculated using Sigmaplot 11.0.

## Figures and Tables

**Table 1:** The cytotoxicity of different compounds in resistant and sensitive cancer cells. The IC_50_ values (μM) of capsaicin, piperine, and doxorubicin against different cell lines are presented as the mean ± SD.

Compounds	Caco-2	HCT 116	CEM/ADR 5000	CCRF-CEM
Doxorubicin	4.97 ± 0.36	0.92 ± 0.07	92.59 ± 7.90	0.37 ± 0.14
Capsaicin	163.70 ± 9.32	66.77 ± 10.78	125.85 ± 22.05	67.55 ± 6.29
Piperine	101.30 ± 6.97	74.30 ± 10.35	121.77 ± 17.35	102.18 ± 6.82
Digitonin	18.39 ± 1.44	9.04 ± 0.73	16.69 ± 2.06	10.34 ± 1.18

**Table 2:** Cytotoxicity of doxorubicin against Caco-2 cells, either alone or in two-drug or three-drug combinations. The IC_50_ (μM) are presented as the mean ± SD. 0.3 < CI < 0.7 means synergism (+ + +), 0.7–0.85 moderate synergism (+ +). NR = not relevant. IB = isobologram. DRI = dose reduction indexes.

	Two-Drug Combinations					Three-Drug Combinations with Digitonin				
	IC_50_ of Dox	IB	DRI	CI	Interpretation	IC_50_ of Dox	IB	DRI	CI	Interpretation
Doxorubicin alone	4.97 ± 0.36		1.00	NR	NR	4.97 ± 0.36		1.00	NR	NR
Dox + Capsaicin										
20 μM (IC_10_)	2.14 ± 0.37	syn	2.33	0.56	+ + +	1.35 ± 0.56	syn	3.68	0.30	+ + +
34 μM (IC_20_)	1.54 ± 0.51	syn	3.22	0.42	+ + +	1.07 ± 0.34	syn	4.66	0.35	+ + +
50 μM (IC_30_)	1.26 ± 0.04	syn	3.94	0.57	+ + +	0.59 ± 0.12	syn	8.39	0.41	+ + +
Dox + Piperine										
30 μM (IC_10_)	2.05 ± 0.39	syn	2.42	0.80	+ +	1.60 ± 0.65	syn	3.11	0.77	+ +
50 μM (IC_20_)	1.52 ± 0.10	syn	3.27	0.81	+ +	1.02 ± 0.13	syn	4.88	0.72	+ +
65 μM (IC_30_)	0.90 ± 0.14	syn	5.52	0.76	+ +	0.32 ± 0.09	syn	15.65	0.63	+ + +

**Table 3:** Cytotoxicity of doxorubicin against CEM/ADR 5000 cells, either alone or in two-drug or three-drug combinations. The IC50 (μM) are presented as the mean ± SD. 0.3 < CI < 0.7 synergism (+ + +), 0.7–0.85 moderate synergism (+ +), 0.85–0.9 slight synergism (+). NR—not relevant. IB—isobologram. DRI—dose reduction indexes.

	Two-Drug Combinations					Three-Drug Combinations with Digitonin				
	IC_50_ of Dox	IB	DRI	CI	Interpretation	IC_50_ of Dox	IB	DRI	CI	Interpretation
Doxorubicin alone	92.59 ± 7.90		1.00	NR	NR	92.59 ± 7.90		1.00	NR	NR
Dox + Capsaicin										
20 μM (IC_10_)	64.73 ± 10.40	syn	1.43	0.86	+	39.87 ± 8.01	syn	2.32	0.59	+ + +
30 μM (IC_20_)	44.17 ± 7.78	syn	2.10	0.72	+ +	28.84 ± 5.65	syn	3.21	0.55	+ + +
50 μM (IC_30_)	31.61 ± 9.56	syn	2.93	0.74	+ +	19.10 ± 7.02	syn	4.85	0.60	+ + +
Dox + Piperine										
25 μM (IC_10_)	62.85 ± 9.65	syn	1.47	0.88	+	23.39 ± 4.28	syn	3.96	0.46	+ + +
30 μM (IC_20_)	45.13 ± 7.92	syn	2.05	0.69	+ + +	21.52 ± 1.02	syn	4.30	0.44	+ + +
45 μM (IC_30_)	41.02 ± 9.27	syn	2.26	0.65	+ + +	8.54 ± 4.55	syn	10.84	0.30	+ + +
